# Sports and Health, Second Edition

**DOI:** 10.3390/ijerph19148435

**Published:** 2022-07-10

**Authors:** Pantelis T. Nikolaidis, Beat Knechtle

**Affiliations:** 1 School of Health and Caring Sciences, University of West Attica, 12243 Athens, Greece; 2 Institute of Primary Care, University of Zurich, 8091 Zurich, Switzerland; beat.knechtle@hispeed.ch

The *International Journal of Environmental Research and Public Health* (*IJERPH*) has increased its publications of scientific papers related to exercise; a search of Pubmed (on 22 June 2022) using *IJERPH* and exercise as keywords showed 1788 entries for 2021 compared to 80 entries in 2016 [1]. This increase over the last five years is characteristic of the actual multi-disciplinary scientific interest in the role of exercise in health. Moreover, the first edition of our Special Issue ‘Sport and Health’ was successful, publishing 52 papers [2], the paper of Lesser and Nienhuis [3] being the most cited one (293 citations within a two-year period). This scientific interest corresponds to an ongoing increase in participation in sports, e.g., 236 men and 8 women competed in the Berlin Marathon in 1974 compared to 28,373 men and 12,268 women in 2018 [4], and reflects a broad interest in examining the relationship between health and exercise. Therefore, the aim of the present Special Issue [5] is to develop an updated article collection on the relationship between health and sports participation across all lifestyles, with an emphasis on recreational athletes, including studies on the role of COVID-19 [6].
